# High-cell-density fed-batch cultivations of *Vibrio natriegens*

**DOI:** 10.1007/s10529-021-03147-5

**Published:** 2021-05-19

**Authors:** Isabel Thiele, Björn Gutschmann, Linus Aulich, Marcel Girard, Peter Neubauer, Sebastian L. Riedel

**Affiliations:** Chair of Bioprocess Engineering, Institute of Biotechnology, Technische Universität Berlin, Berlin, Germany

**Keywords:** High-cell-density cultivations, Fed-batch, *Vibrio natriegens*, Fast growing microorganism, Parallel bioreactor cultivations, Deep-well plate

## Abstract

**Objectives:**

With generation times of less than 10 min under optimal conditions, the halophilic *Vibrio natriegens* is the fastest growing non-pathogenic bacterium isolated so far. The availability of the full genome and genetic engineering tools and its ability to utilize a wide range of carbon sources make *V. natriegens* an attractive host for biotechnological production processes. However, high-cell-density cultivations, which are desired at industrial-scale have not been described so far.

**Results:**

In this study we report fed-batch cultivations of *V. natriegens* in deep-well plates and lab-scale bioreactor cultivations at different temperatures in mineral salt medium (MSM). Upon switching from exponential glucose to constant glucose-feeding cell death was induced. Initial NaCl concentrations of 15–18 g L^−1^ and a temperature reduction from 37 to 30 °C had a positive effect on cell growth. The maximal growth rate in MSM with glucose was 1.36 h^−1^ with a specific oxygen uptake rate of 22 mmol g_CDW_^−1^ h^−1^. High biomass yields of up to 55 g L^−1^ after only 12 h were reached.

**Conclusions:**

The shown fed-batch strategies demonstrate the potential of *V. natriegens* as a strong producer in industrial biotechnology.

**Supplementary Information:**

The online version of this article (doi:10.1007/s10529-021-03147-5) contains supplementary material, which is available to authorized users.

## Introduction

Only recently the potential of *Vibrio natriegens* (formerly known as *Pseudomonas natriegens* and *Beneckea natriegens*) has been rediscovered for biotechnological applications. The halophilic Gram-negative γ-proteobacterium was first isolated from salt marsh mud in 1961 (Eagon 1961) and is still the fastest-growing non-pathogenic bacterium reported so far (Long et al. 2017). *Vibrio natriegens* requires Na^+^, Mg^2+^ and K^+^ for the induction of enzymes involved in substrate uptake and utilization for its outstandingly fast growth (Eagon 1961; Rhodes and Payne 1962). Similar to the model organism *Escherichia coli*, it is facultatively anaerobic, prefers neutral pH, has an optimal growth temperature of 37 °C and shows a similar core metabolism under aerobic growth on glucose (Long et al. 2017). However, in complex media *V. natriegens* has a generation time of only 9.4 min (growth rate *µ* = 4.43 h^−1^), which is less than half of that of *E. coli* and other important model organisms such as *Bacillus subtilis* even under optimal conditions (Eagon 1961; Hecker and Völker 1990; Sezonov et al. 2007; Hoffart et al. 2017).

While there exists a variety of synthetic biology tools for *V. natriegens*, including those for genetic and metabolic engineering, that allow for transformation efficiencies similar to *E. coli*, cultivation strategies to achieve very high-cell densities favorable to industrial processes have not been well reported in the literature so far (Weinstock et al. 2016; Dalia et al. 2017; Hoffart et al. 2017; Schleicher et al. 2018; Eichmann et al. 2019; Lee et al. 2019; Tschirhart et al. 2019). Fed-batch cultivations can be used to obtain high-cell-densities by controlled supply of the carbon source. Due to a controlled growth rate at carbon limitation, undesired byproducts might be omitted and requirements like adequate levels of dissolved oxygen (DO) are met, which might be limited in large-scale processes.

In this study, we report the use of small scale deep-well sensor plates to screen for optimal *V. natriegens* growth conditions and parallelized fed-batch bioreactors to achieve high-cell-densities at laboratory benchtop-scales.

## Materials and methods

### Bacterial strain and chemical materials

*Vibrio natriegens* ATCC 14048 (wild type, DSM 759) from the German collection of microorganisms and cell cultures GmbH was used for all experiments. Chemicals were purchased from Sigma Aldrich (USA), Carl Roth GmbH & Co. KG (Germany), Merck KGaA (Germany) or VWR Chemicals (USA).

### Growth media and preculture conditions

100 µL of a *V. natriegens* cryostock stored at − 80 °C were used to inoculate a 125-mL shake flask containing 10 mL LB medium (10 g L^−1^ tryptone, 5 g L^−1^ yeast extract, 15 g L^−1^ NaCl, pH 7.5), which was cultivated at 37 °C and 200 rpm at 25 mm amplitude for 3–4 h and used to inoculate the main cultures while in the exponential growth phase at an optical density at 600 nm (OD_600_) of the preculture of about 1.5.

### Development of small-scale culture conditions

Parallel cultivations in 24 deep-well-sensor plates (PreSens Precision Sensing GmbH, Regensburg, Germany) with different NaCl concentrations and buffer conditions were performed using the instant fed-batch medium EnPresso B® (Enpresso GmbH, Berlin, Germany), which allows for an automatic, enzyme based, glucose limited fed-batch cultivation in small-scale. The 24 deep-well, flat bottom HydroDish® (HD24) and OxoDish® (OD24) plates are sterile, single use plates, equipped with pre-calibrated pH and DO sensors, respectively, to measure pH and DO *on-line*. The working volume was 1 mL. The cultures were incubated at 37 °C in an orbital shaker (25 mm amplitude, Infors HT, Bottmingen, Switzerland) for 24 h at 300 rpm. The tested conditions included six levels of NaCl concentrations, ranging from 0 to 24 g L^−1^ with the supplementation of 24 g L^−1^ 3-(*N*-morpholino)propane sulfonic acid (MOPS). Additionally, 0 and 12 g L^−1^ NaCl were also tested without MOPS supplementation. Each 1 mL culture contained 0.1 µL antifoam (PPG2000). The experiments were performed in triplicates on each plate, i.e. six aliquots of each cultivation condition.

### Bioreactor fed-batch cultivations

Fed-batch cultivations were performed using mineral salt medium (MSM), which was sterilized in the bioreactors at 121 °C for 20 min, consisting of 15 g L^−1^ NaCl, 5 g L^−1^ (NH_4_)_2_SO_4_, 1 g L^−1^ KH_2_PO_4_ and 1 g L^−1^ K_2_HPO_4_. The medium was supplied with separately autoclaved glucose solution (20 min, 121 °C) and sterile filtered MgSO_4_ × 7 H_2_O solution (0.2 µM CA filter) to obtain a final concentration of 10 g L^−1^ glucose and 0.5 g L^1^ MgSO_4_ × 7 H_2_O. To complete the medium 1 % (v v^−1^) of separately sterile filtered 100 × trace element solution composed of: 1.64 g L^−1^ FeSO_4_ × 7 H_2_O, 1.35 g L^−1^ CaCO_3_ × 2 H_2_O, 1.0 g L^−1^ MnSO_4_ × H_2_O, 0.1 g L^−1^ ZnSO_4_ × 7 H_2_O, 0.03 g L^−1^ CuSO_4_ × 5 H_2_O, 0.002 g L^−1^ NiCl_2_ × 6 H_2_O was added. The pH was adjusted to 7.50 by addition of 3 M NaOH or 1 M H_3_PO_4_. The feeding solution consisted of 600 g L^−1^ glucose, 15 g L^−1^ NaCl, 20 g L^−1^ (NH_4_)_2_SO_4_, 20 g L^−1^ KH_2_PO_4_, 20 g L^−1^ K_2_H_2_PO_4_, 5 g L^−1^ MgSO_4_ and 1% (v v^−1^) trace element solution, as well as 0.01% (v v^−1^) PPG2000 as an antifoaming agent.

Fed-batch cultivations were performed in parallel benchtop stirred tank reactors with 1 L working volume (Multifors 2, Infors AG, Bottmingen, Switzerland). The temperature was set to 30 or 37 °C, respectively. The pH value was maintained at 7.50 (± 0.05) by addition of 3 M NaOH or 1 M H_3_PO_4_. The bioreactors were equipped with two six-blade Rushton impellers. The initial stirring speed and air flow were set to 200 rpm and 0.5 vvm, respectively. In order to maintain the DO concentration above 40% during the cultivation an automated cascade consisting of an increased stirrer speed (200–1500 rpm), increased air flow (0.5–2 vvm) and at last increased gas mix of oxygen in the supplied air (0–70%), was used. Foam breakers made from cable ties were attached at the top of the stirrer shaft. Additionally, pulses of 0.1 mL of antifoam (PPG2000) where added manually whenever needed.

The fed-batch cultivations were started with an initial batch phase in 500 mL medium and the cultures were inoculated to an initial OD_600_ of 0.15. After glucose depletion, the feed solution was fed exponentially according to Eq. 1. The specific growth rate (*µ*_set_) was set to 50% of *µ*_max_.

1 $$F\left(t\right)={F}_{0}*{e}^{{\mu }_{set}*t}.$$

The initial feed rate *F*_0_ (g h^−1^) was calculated using Eq. 2. The biomass concentration (*X*) was estimated from a previously correlation of OD_600_ with cell dry weight (CDW) values. The biomass/substrate yield *Y*_X/S_ was calculated from the batch phase with the initial glucose concentration (*S*). *S*_i_ represents the glucose concentration of the feed solution and *X*_0_ and *V*_0_ are the biomass concentration and culture volume at the beginning of the fed-batch, respectively.

2 $${F}_{0}=\frac{{\mu }_{set}}{{Y}_{X/S}*{S}_{i}}\left({X}_{0}{V}_{0}\right).$$

After 4 h of exponential feeding, the cultivation was continued for another 2 h with constant feeding at the last rate of the exponential feed.

### Sampling and analytical methods during fed-batch cultivations

Samples of the culture broth were taken every hour to determine OD_600_ *off-line* in technical duplicates by using a standard UV cuvette spectrophotometer. Furthermore, 2 mL aliquots were filled into pre-weighed 2 mL reaction tubes for CDW determination in triplicates. The samples were centrifuged at 16,000×*g* at 4 °C for 10 min and the pellet was washed under the same conditions with 0.9% NaCl before it was dried at 80 °C. The supernatants were analyzed with the Cedex Bio HT Analyzer® (Roche Diagnostics International AG, Rotkreuz, Switzerland) using test kits for glucose, ammonia, acetate, ethanol, formate and lactate concentration (Glucose Bio HT, NH3 Bio HT, Acetate V2 Bio HT, Ethanol Bio HT, Formate Bio HT and Lactate Bio HT).

Exhaust gas data was recorded for one reactor for each temperature (30 or 37 °C) during the fed-batch cultivation and served for the determination of the oxygen uptake rate (*OUR*, $Q_{{\text{O}}_{2}}$), carbon dioxide production rate ($Q_{{\text{CO}}_{2}}$), respiration coefficient (*RQ*) and the volumetric oxygen transfer coefficient (*k*_*L*_*a*) via gas mass balance.

## Results

### Deep-well plate cultivations

**Fig. 1:**
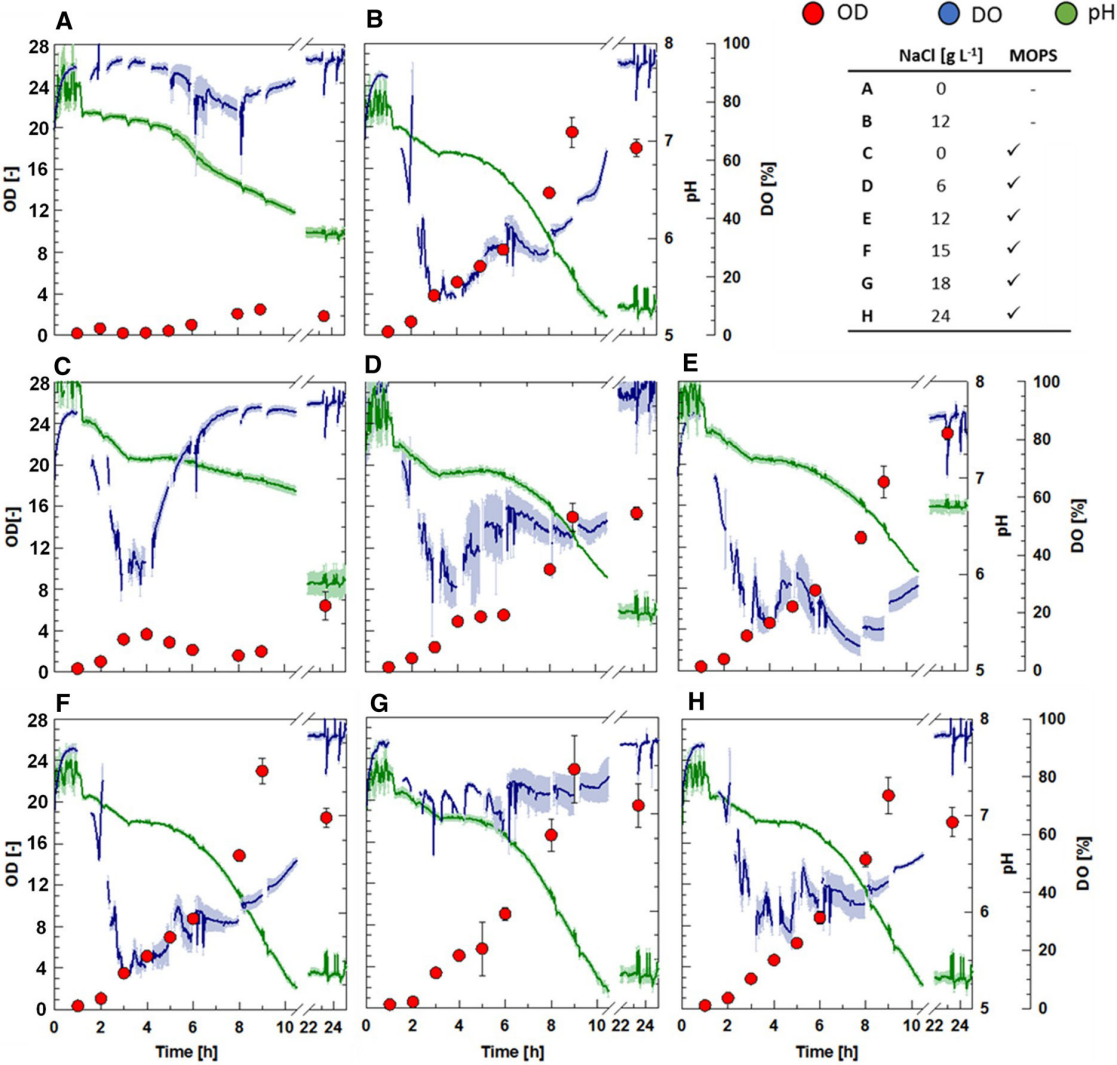
Deep-well plate cultivations of *Vibrio natriegens* in EnPresso B® medium supplemented with varying NaCl and 3-(*N*-morpholino)propane sulfonic acid (MOPS) amounts. The following amounts of NaCl were used (g L^−1^): **a** 0, **b** 12, **c** 0, **d** 6, **e** 12, **f** 15, **g** 18, and **h** 24. **a** and **b** were not supplemented with MOPS, whereas 24 g L^−1^ MOPS were added to **c**–**h**. The dissolved oxygen (DO) concentration and pH were monitored by using HydroDish® and OxoDish® plates, respectively. DO and pH data point are mean values from biological triplicates and OD_600_ data points represent mean values of six biological replicates. Error bars indicate ± SD

To evaluate *V. natriegens*’ response to different cultivation conditions DO and pH were monitored *on-line*. The cultures were grown in EnPresso B medium supplemented with different NaCl concentrations and with an optional addition of MOPS buffer (Fig. 1). During the first 2 h data indicates a short lag phase in all cultures. The pH decreased from an initial value of 7.5 rapidly to 7.0 within the first 4 h, resembling typical growth behavior in batch cultivations due to ammonia uptake. In the following 2 h the pH remained stable with a subsequent rapid drop to 5.2–5.8 depending on the corresponding OD_600_ (Fig. 1). After 24 h no further growth was detected. The absence of NaCl results in slow growth and low OD_600_ values below 2 after 10 h of cultivation (Fig. 1a, c). OD_600_ after 1 day increased in the culture with additional MOPS buffer (Fig. 1c) while no buffer addition led to a decrease in the OD_600_ (Fig. 1a). Between 4 and 6 h DO consumption is highest and correlates with fast cell growth. NaCl concentrations of about 12–18 g L^−1^ achieved higher OD_600_ of 20–24 compared to lower NaCl concentrations after 10 h of cultivation (Fig. 1e–g).

### Fed-batch cultivations

**Fig. 2:**
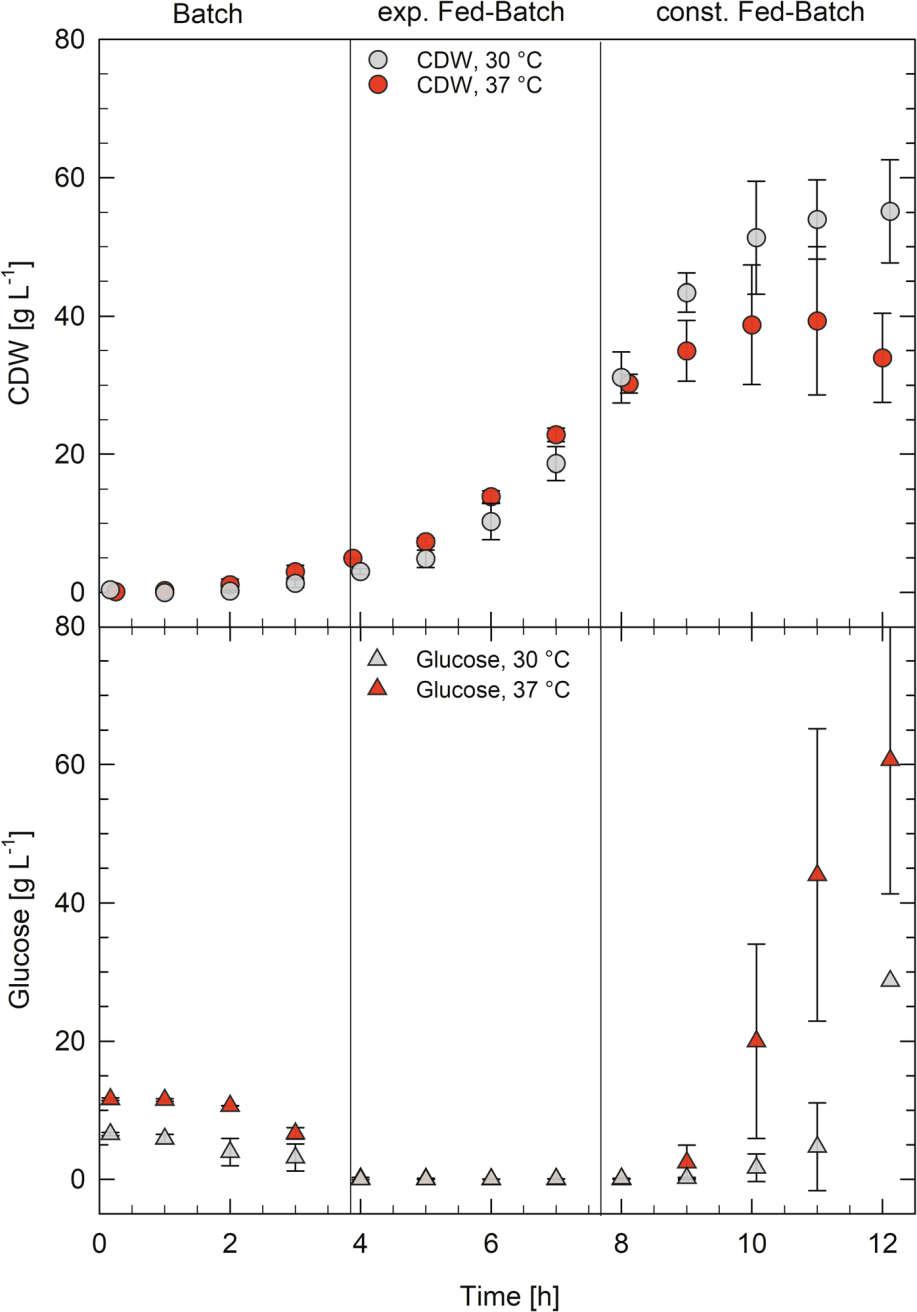
Glucose limited fed-batch cultivations of *Vibrio natriegens* at 30 °C (gray) and 37 °C (red). Cell dry weight (CDW) and glucose concentrations are shown. Data points are mean values of triplicate bioreactor cultivations. Error bars indicate ± SD

To achieve high-cell-densities, glucose limited fed-batch cultivations were performed in 1-L stirred tank bioreactors at 37 and 30 °C, respectively. The exponential slope of glucose consumption corresponds to the exponential growth behavior during the batch phase. Maximum specific growth rates (*µ*_max_) of 0.79 h^−1^ at 30 °C and 1.36 h^−1^ at 37 °C were determined during the batch phase. Glucose depletion was monitored to observe the end of the batch phase after ~ 4 h of cultivation. Exponential feeding with 60% (w v^−1^) glucose solution (feed medium) proceeded for another 4 h according to Eq. 1. Afterwards a constant feed phase of 2 h followed. Biomass yield coefficient (*Y*_X/S_) after the batch phase was 0.36 g g^−1^ at 37 °C and 0.71 g g^−1^ at 30 °C with a *q*_s_ of 2.18 and 1.86 g g_CDW_^−1^ h^−1^, respectively. During the controlled exponential feed, a growth rate of 0.58 h^−1^ at 30 °C and of 0.42 h^−1^ at 37 °C was obtained. In the constant feeding phase, the cultures at 30 °C accumulated a higher CDW of 55 g L^−1^ than the cultures at 37 °C, which had decreased to 34 g L^−1^ until the end of the cultivation at 12 h (Fig. 2).

#### Formation of side products

**Fig. 3:**
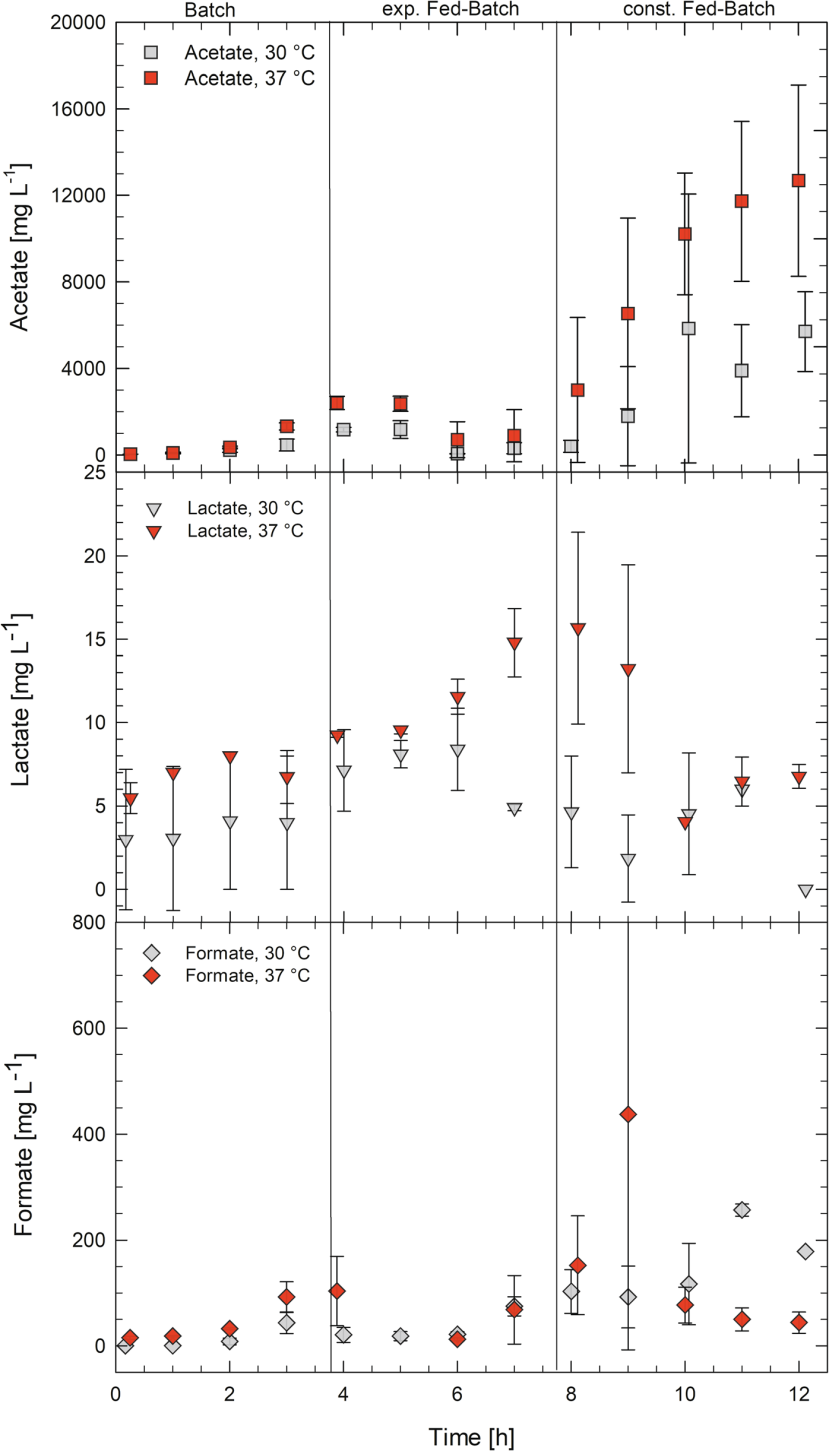
Production of acetate, lactate, and formate during the fed-batch cultivation of *Vibrio natriegens* at 30 and 37 °C. Data points are obtained from triplicate bioreactor cultivations. Error bars indicate ± SD

**Fig. 4:**
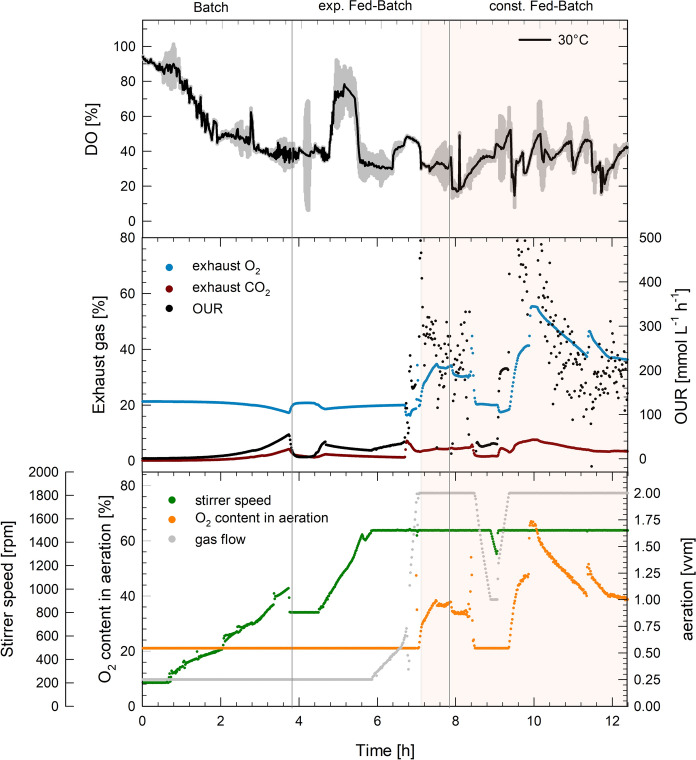
Process data of *Vibrio natriegens* cultivations at 30 °C. Average course of the dissolved oxygen concentration (DO) (%) ± SD (shaded area) during triplicate fed-batch cultivations (upper graph). Exhaust O_2_ and CO_2_ (%) in the exhaust gas and oxygen uptake rate OUR (mmol L^−1^ h^−1^) of one reactor are shown in the middle graph, as well as the respective stirrer speed, oxygen content in the inlet air and aeration (lower graph). The orange shaded area represents the phase where technical oxygen was added to the supplied air

The most abundant side product in the bioreactor cultivations was acetate, that accumulated during the batch phase while glucose was available in excess. Formation of acetate matched *q*_s_ at each temperature with differently fast growth rates. Acetate produced by *V. natriegens* during the batch phase was consumed again in the exponential fed-batch phase. In the subsequent constant feeding phase acetate accumulated again (Fig. 3). Although, no DO limitation occurred during the cultivations (Fig. 4), lactate and formate were produced at both cultivation temperatures. The lactate concentration decreased in the fed-batch phase at 30 °C, whereas it increased during cultivations at 30 °C. The formate formation stayed constant for both temperatures during the feeding phase (Fig. 4).

#### Oxygen uptake rates

**Fig. 5:**
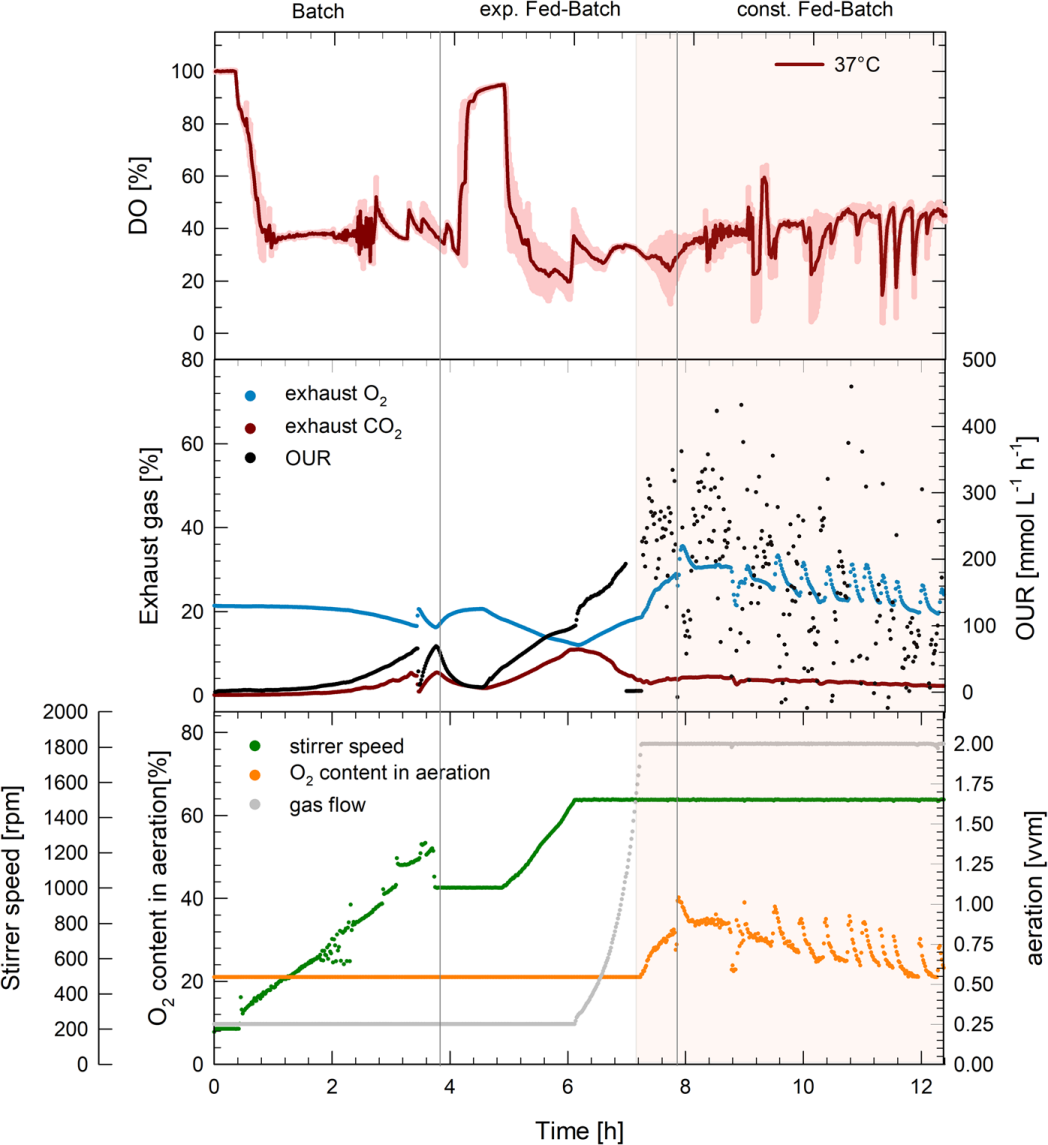
Process data of *Vibrio natriegens* cultivations at 37 °C. Average course of the dissolved oxygen concentration (DO) (%) ± SD (shaded area) during triplicate fed-batch cultivations (upper graph). Exhaust O_2_ and CO_2_ (%) in the exhaust gas and oxygen uptake rate OUR (mmol L^−1^ h^−1^) of one reactor are shown in the middle graph, as well as the respective stirrer speed, oxygen content in the inlet air and aeration (lower graph). The orange shaded area represents the phase where technical oxygen was added to the supplied air

The *OUR* during fed-batch cultivations remained mainly between 100 and 300, but maximum values of up to 500 mmol L^−1^ h^−1^ were reached during the exponential feeding phase (Figs. 4, 5). The high oxygen demand was met by acceleration of the stirrer speed, whenever a critical DO of 40% was reached. With increasing biomass, the gas flow was increased up to 2 vvm and supplied with up to 60% technical oxygen. This way, maximum *k*_*L*_*a* values of about 1600 h^−1^ and 1400 h^−1^ at 37 and 30 °C were obtained, respectively (Supplemental Fig. 1). In the batch phase, the specific oxygen uptake rate ($q_{{\text{O}}_{2}}$) was between 22 and 23 mmol g_CDWw_^−1^ h^−1^ at 37 °C and between 11 and 13 mmol g_CDW_^−1^ h^−1^ at 30 °C. Despite the different growth rates, $q_{{\text{O}}_{2}}$ was comparable with 6–9 mmol g_CDW_^−1^ h^−1^ at both temperatures during the exponential feeding phase (Table 1).

**Table 1:** Specific oxygen uptake rates during the batch and exponential fed-batch phase of the cultivations of *Vibrio natriegens* at 30 and 37 °C

	$q_{{\text{O}}_{2},\,{\text{batch}}}$ (mmol g_CDW_^−1^ h^−1^)	$q_{{\text{O}}_{2},\,{\text{exp.\,fed-batch}}}$ (mmol g_CDW_^−1^ h^−1^)
30 °C	11–13	7–9
37 °C	22–23	6–9

## Discussion

High-cell-densities of *V. natriegens* by fed-batch cultivation using 1-L stirred tank bioreactors were shown for the first time. The aerobic cultivation included an initial batch phase, followed by a glucose-limiting exponential and a subsequent constant feeding phase. So far only batch, pulse based fed-batch or chemostat cultivations with cell density below 10 g L^−1^ CDW have been described for *V. natriegens* (Rhodes and Payne 1962; Long et al. 2017; Schleicher et al. 2018; Becker et al. 2019; Erian et al. 2020). The observed maximum growth rate of 1.36 h^−1^ during the batch phase of the 37 °C bioreactor cultivations were comparable to the maximum growth rates in recent literature under similar conditions (Hoffart et al. 2017; Long et al. 2017). Specific oxygen uptake rates of 22 mmol g_CDW_^−1^ h^−1^ of *V. natriegens* at 37 °C during the batch phase were comparable to the results obtained by Long et al., who obtained a $q_{{\text{O}}_{2}}$ of 28 mmol g_CDW_^−1^ h^−1^ at 37 °C in minimal medium supplied with glucose and a growth rate of 1.7 (although complex media components were used) (Long et al. 2017). During the fed-batch cultivation high OURs of around 300, up to 500 mmol L^−1^ h^−1^ were observed. Such high oxygen demand is unfavorable for industrial scale cultivations due to limited power input. However, tailored aeration systems, e.g. nozzles, could be applied to achieve transfer efficiencies of almost 100% and oxygen transfer rates of 500 mmol L^−31^ h^−1^ (Noorman et al. 2018).

Towards the end of the fed-batch cultivation at 37 °C a CDW reduction from 39 to 34 g L^−1^ was observed, whereas the CDW could be increased up to 55 g L^−1^ during cultivations at 30 °C (Fig. 2). The decrease in biomass during the 37 °C cultivations may arise from the spontaneous activation of prophages. Pfeifer et al. identified two inducible prophage regions on the first chromosome of *V. natriegens* ATCC 14048 (wild type, DSM 759), the same strain as used in this study (Pfeifer et al. 2019). Prophage induction was shown to happen spontaneously under standard cultivation conditions (Wiegand et al. 2018).

The instant medium EnPresso B (based on EnBase™ technology; Panula-Perälä et al. 2008), which enables a glucose limited fed-batch in mL-scale was used during deep-well plate cultivations. However, the growth behavior was not typical for EnPresso B cultivations, since no linear growth phase, through the enzyme controlled linear glucose release from starch, was visible after the batch phase (Fig. 1). *Vibrio natriegens* can utilize and hydrolyze a wide range of carbon sources (Hoffart et al. 2017; Ellis et al. 2019; Tschirhart et al. 2019). Therefore *V. natriegens* grew independent from the released glucose from the starch of the EnPresso B medium. However, no DO limitation occurred during these cultivations, indicating a slower growth on starch as on glucose. Consumption of the glucose excess in the EnPresso B medium was visible through an increase of the DO concentration (Fig. 1).

To cope with the outstanding growth rates and fast metabolism of *V. natriegens*, suitable fermentation processes must be developed, to prevent the formation of overflow metabolites, which are reducing the biomass yield and become toxic with increasing concentration (Neubauer et al. 2003). The production of mixed acid fermentation products was investigated in aerobic fed-batch cultivations at 30 and 37 °C. Interestingly, formate and lactate, beside acetate formation was obtained although no DO limitation occurred (Figs. 3, 4), while previously studies only observed acetate formation during aerobic batch cultivations (Hoffart et al. 2017; Long et al. 2017). Lowering the cultivation temperature from 37 to 30 °C only led to a slightly lower formation of acetate, lactate and formate (Fig. 3). However, the total biomass could thereby be increased by 60%, from 34 to 55 g L ^1^, which resulted in a biomass productivity of 4.6 g L^−1^ h^−1^.

Pre-experiments in deep-well plates confirmed an ideal initial NaCl concentration of 15–18 g L^−1^ for aerobic *V. natriegens* cultivations (Fig. 1) (Hoffart et al. 2017; Long et al. 2017). The high NaCl demand of *V. natriegens* could be problematic in industrial applications due to Cl^−^ speeding up corrosion of stainless steel bioreactors. However, the usage of high NaCl containing media can also allow for semi or even unsterile cultivation conditions, which reduces energy costs during industrial production processes (Yin et al. 2015). Also, with the trend going to different single use reactor systems, like orbital shaken bag reactors, corrosion might not even be a problem anymore (Oosterhuis et al. 2013).

## Conclusions

High-cell-density fed-batch cultivations of *V. natriegens* were performed at deep-well- to bioreactor-scale and resulted in a maximum biomass productivity of 4.6 g L^−1^ h^−1^ with a total CDW of 55 g L^−1^ after 12 h, in a glucose-limited fed-batch with 1.5% NaCl at 30 °C. The feeding strategy and a lower cultivation temperature led to less side products and higher biomass concentrations. The feasibility of a fed-batch cultivation of *V. natriegens* further highlights the organism’s potential for industrial application and the future prospects to combine with the already available biotechnological tools to become an alternative production organism for e.g. recombinant protein production.

## Electronic supplementary material

Below is the link to the electronic supplementary material.

(DOCX 580 kb)

## References

[CR1] Becker W, Wimberger F, Zangger K (2019). *Vibrio natriegens*: an alternative expression system for the high-yield production of isotopically labeled proteins. Biochemistry.

[CR2] Dalia TN, Hayes CA, Stolyar S (2017). Multiplex genome editing by natural transformation (MuGENT) for synthetic biology in *Vibrio natriegens*. ACS Synth Biol.

[CR3] Eagon RG (1961). *Pseudomonas natriegens*, a marine bacterium with a generation time of less than 10 minutes. J Bacteriol.

[CR4] Eichmann J, Oberpaul M, Weidner T (2019). Selection of high producers from combinatorial libraries for the production of recombinant proteins in *Escherichia coli* and *Vibrio natriegens*. Front Bioeng Biotechnol.

[CR5] Ellis GA, Tschirhart T, Spangler J (2019). Exploiting the feedstock flexibility of the emergent synthetic biology chassis *Vibrio natriegens* for engineered natural product production. Mar Drugs.

[CR6] Erian AM, Freitag P, Gibisch M, Pflügl S (2020). High rate 2,3-butanediol production with *Vibrio natriegens*. Bioresour Technol Rep.

[CR7] Hecker M, Völker U (1990). General stress proteins in *Bacillus subitlis*. FEMS Microbiol Ecol.

[CR8] Hoffart E, Grenz S, Lange J (2017). High substrate uptake rates empower *Vibrio natriegens* as production host for industrial biotechnology. Appl Environ Microbiol.

[CR9] Lee HH, Ostrov N, Wong BG (2019). Functional genomics of the rapidly replicating bacterium *Vibrio natriegens* by CRISPRi. Nat Microbiol.

[CR10] Long CP, Gonzalez JE, Cipolla RM, Antoniewicz MR (2017). Metabolism of the fast-growing bacterium *Vibrio natriegens* elucidated by ^13^C metabolic flux analysis. Metab Eng.

[CR11] Neubauer P, Lin HY, Mathiszik B (2003). Metabolic load of recombinant protein production: inhibition of cellular capacities for glucose uptake and respiration after induction of a heterologous gene in *Escherichia coli*. Biotechnol Bioeng.

[CR12] Noorman HJ, van Winden W, Heijnen JJ, van der Lans RGJM, Górak A, Stankiewicz A, Siedl P (2018). Intensified fermentation processes and equipment. Intensification of biobased processes.

[CR13] Oosterhuis NM, Neubauer P, Junne S (2013). Single-use bioreactors for microbial cultivation. Pharm Bioprocess.

[CR14] Panula-Perälä J, Šiurkus J, Vasala A (2008). Enzyme controlled glucose auto-delivery for high cell density cultivations in microplates and shake flasks. Microb Cell Factories.

[CR15] Pfeifer E, Michniewski S, Polen T (2019). Generation of a prophage-free variant of the fast-growing bacterium *Vibrio natriegens*. Appl Environ Microbiol.

[CR16] Rhodes ME, Payne WJ (1962). Further observations on effects of cations on enzyme induction in marine bacteria. Antonie Van Leeuwenhoek.

[CR17] Schleicher L, Muras V, Claussen B (2018). *Vibrio natriegens* as host for expression of multisubunit membrane protein complexes. Front Microbiol.

[CR18] Sezonov G, Joseleau-Petit D, D’Ari R (2007). *Escherichia coli* physiology in Luria–Bertani broth. J Bacteriol.

[CR19] Tschirhart T, Shukla V, Kelly EE (2019). Synthetic biology tools for the fast-growing marine bacterium *Vibrio natriegens*. ACS Synth Biol.

[CR20] Weinstock MT, Hesek ED, Wilson CM, Gibson DG (2016). *Vibrio natriegens* as a fast-growing host for molecular biology. Nat Methods.

[CR21] Wiegand DJ, Lee HH, Ostrov N, Church GM (2018). Establishing a cell-free *Vibrio natriegens* expression system. ACS Synth Biol.

[CR22] Yin J, Chen JC, Wu Q, Chen GQ (2015). Halophiles, coming stars for industrial biotechnology. Biotechnol Adv.

