# Supplementary material for: High-cell-density fed-batch cultivations of Vibrio natriegens
Source: Biotechnol Lett. 2021 May 19;43(9):1723–33. doi: 10.1007/s10529-021-03147-5 (PMC8397650; doi:10.1007/s10529-021-03147-5)
Supplement: Supplementary file 1 — (DOCX 580 kb) [file 10529_2021_3147_MOESM1_ESM.docx]

*Supplemental material*

*Original research paper*

**High-cell-density fed-batch cultivations of *Vibrio*** ***natriegens***

**Isabel Thiele ^1^, Björn Gutschmann ^1^, Linus Aulich ^1^, Marcel Girard ^1^, Peter Neubauer ^1^, Sebastian L. Riedel ^1,^ ***

^1^ Technische Universität Berlin, Institute of Biotechnology, Chair of Bioprocess Engineering, Berlin, Germany

*Correspondence: [riedel@tu-berlin.de](mailto:riedel@tu-berlin.de)

**Supplemental Figure legends**

**Supplemental Figure 1** Exhaust gas analysis of *Vibrio natriegens* fed-batch cultivations at 30 and 37°C. The volumetric oxygen transfer coefficient *k_L_a* [h^-1^], oxygen uptake rate (*Q*_O2_) and carbon dioxide production rate (*Q*_CO2_) [mol L^-1^ h^-1^], as well as respiration coefficient (*RQ*) [ - ] were determined using gas a mass balance of exhaust gas data. The orange shaded area represents the phase where technical oxygen was added to the supplied air.

**Supplemental Figures**

**Supplemental Figure 1**

**

**
